# BrainNet: a fusion assisted novel optimal framework of residual blocks and stacked autoencoders for multimodal brain tumor classification

**DOI:** 10.1038/s41598-024-56657-3

**Published:** 2024-03-11

**Authors:** Muhammad Sami Ullah, Muhammad Attique Khan, Nouf Abdullah Almujally, Majed Alhaisoni, Tallha Akram, Mohammad Shabaz

**Affiliations:** 1 Department of Computer Science, HITEC University, Taxila, Pakistan; 2 Department of Computer Science and Mathematics, Lebanese American University, Beirut, Lebanon; 3 Department of Computer Science, HITEC University, Taxila, 47080 Pakistan; 4 Department of Information Systems, College of Computer and Information Sciences, Princess Nourah Bint Abdulrahman University, PO Box 84428, 11671 Riyadh, Saudi Arabia; 5 Computer Sciences Department, College of Computer and Information Sciences, Princess Nourah Bint Abdulrahman University, Riyadh, Saudi Arabia; 6 Department of ECE, COMSATS University Islamabad, Wah Campus, Rawalpindi, Pakistan; 7 Model Institute of Engineering and Technology, Jammu, J&K India

**Keywords:** Brain tumor, Explinable AI, MRI, Residual blocks, Stack encoders, Feature selection, Fusion, Classification, Medical research, Neurology

## Abstract

A significant issue in computer-aided diagnosis (CAD) for medical applications is brain tumor classification. Radiologists could reliably detect tumors using machine learning algorithms without extensive surgery. However, a few important challenges arise, such as (i) the selection of the most important deep learning architecture for classification (ii) an expert in the field who can assess the output of deep learning models. These difficulties motivate us to propose an efficient and accurate system based on deep learning and evolutionary optimization for the classification of four types of brain modalities (t1 tumor, t1ce tumor, t2 tumor, and flair tumor) on a large-scale MRI database. Thus, a CNN architecture is modified based on domain knowledge and connected with an evolutionary optimization algorithm to select hyperparameters. In parallel, a Stack Encoder–Decoder network is designed with ten convolutional layers. The features of both models are extracted and optimized using an improved version of Grey Wolf with updated criteria of the Jaya algorithm. The improved version speeds up the learning process and improves the accuracy. Finally, the selected features are fused using a novel parallel pooling approach that is classified using machine learning and neural networks. Two datasets, BraTS2020 and BraTS2021, have been employed for the experimental tasks and obtained an improved average accuracy of 98% and a maximum single-classifier accuracy of 99%. Comparison is also conducted with several classifiers, techniques, and neural nets; the proposed method achieved improved performance.

## Introduction

An abnormal cell growth developed in the brain is a brain tumor^1^. The microscopic organization of the tissues and distinctive molecular properties of brain tumors serve as classification criteria^2^. The medical team can better forecast a brain tumor's behavior, choose the best course of therapy, and select the potential candidates for clinical trials by knowing the kind and grade of the tumor^3^. According to the World Health Organization (WHO), there are almost 120 types of brain tumors. Each category is further subdivided into two general categories: benign and malignant. According to WHO, 700,000 Americans are surviving with primary brain tumors, whereas 71% have benign tumors and the rest have malignant tumors^4^. The benign tumor is considered a non-cancerous tumor. This kind of tumor does not spread to other parts of the body. They remain in their original location and are usually not problematic. They often have clear borders and a modest rate of growth. Usually, this kind of tumor is small, slow-growing, noninvasive, well-differentiated, and confined^5^. The malignant types of tumors are considered as cancerous. The tumor growth rate is quick; quick growth crowds and disturbs healthy cells. New tumors are created when abnormal cells move through the blood or lymph from one organ to another. Secondary tumors are the name given to these new tumors. In a nutshell, we may say that these tumors are big, aggressive, metastasize, rapidly develop, and are poorly differentiated from their surroundings^6,7^.

In the United States, for a total of 431,773 incident tumors, the incidence rate for all primary malignant and benign brain and other Central Nervous System (CNS) tumors in the US was 24.23 instances per 100,000 (7.06 per 100,000 for malignant tumors (125,524 cases) and 17.18 per 100,000 for non-malignant tumors (306,249 cases)). Females (26.95 per 100,000) had a greater rate than males (21.35 per 100,000)^8^. Brain tumors can be fatal, seriously influence the quality of life, and completely alter a patient's and their loved ones' lives. They affect people of all racial and ethnic backgrounds and do not discriminate^4^.

Brain tumors are diagnosed using different types of brain scans. These brain scans provide useful information about brain tumor location, size, and volume across the brain. Subsequent are the two important diagnostic tools used to diagnose and figure out the tumorous area of the subject. The first one is Computerized Tomography (CT) Scan^9^. It is a noninvasive diagnostic imaging process that creates horizontal or axial brain images using certain X-ray measurements. Compared to ordinary head X-rays, brain CT scans can offer more precise details about brain tissue and structures, revealing more information about the brain, e.g., hydrocephalus, brain tumor, etc.^10^. CT technology produced anatomical images with exceptional clinical detail and transformed medical radiology^11^. The second one is Magnetic Resonance Imaging (MRI), a noninvasive medical imaging procedure that creates precise images of every internal body structure, including the organs, bones, muscles, and blood arteries^12^. Due to its ubiquitous accessibility and capacity to distinguish between soft and hard tissues, MRI is regarded as a standard method and offers extensive information about the anatomy of human tissues. A strong magnetic field and radio frequency signals are used in MRI to create images of human tissues^13,14^. MRI scanners produce images of the human body using a powerful magnet and radio waves.

In contrast to X-rays, no radiation is produced during an MRI exam. Compared with CT technology, the MRI performed better in brain tumor detection^15^. Furthermore, MRI provides improved soft tissue contrast compared to conventional cross-sectional imaging methods, enabling better detection of modestly infiltrated or damaged parenchymal architecture^16^. MRI's features make it an ultimate choice for brain imaging for tumor detection. Since MRI images are based on shape and texture information, they are better than CT images^17^. The prevalence of computer vision in radiology is so significant that it has swiftly developed into a separate field of study, building a body of work^18^. Researchers and physicians now agree that deep learning will play a vital role in radiology^19^. By fusing deep learning and human intelligence, the use of deep learning in radiology will increase the capabilities of the radiologist^20^. The therapy of brain tumors depends on accurate detection, yet manual identification is time-consuming. Deep learning efficiency can only be doubled using a deep learning model^21^. The most accurate way to determine the precise type of brain tumor is to do a biopsy, an intrusive procedure. Biopsies classify tumors based on the microscopic similarities of the cells and their degrees of differentiation. The procedure is invasive and carries additional health concerns. MRI-based noninvasive techniques are being developed as a reliable substitute. Machine learning has been used to differentiate among various tumor types using MRI data. Convolutional Neural Networks (CNN) have also been used to classify brain tumors using deep learning on MRI data^22,23^.

Several challenges have been faced by computer vision researchers using deep learning techniques for brain tumor modalities classification. However, these techniques facing several challenges such as high false positive rate, high similarity rate of healthy and malignant tumor images, redundant features, and less information (features) for the classification. In addition, the selection of pre-trained models such as Resnet is a challenging due to high number of parameters. Also, the features from the single source are not enough to compute the maximum accuracy; but this process increased the redundant information that increased the computational time. Feature optimization algorithms are used to decrease overfitting problem by minimizing the redundant features obtained from previous steps. This means final feature selection will not be based on the noisy data. Noisy features usually mislead the training algorithm and leads to the loss of overall accuracy. To resolve these challenges, a new framework is proposed for the classification of brain tumor using MRI scans based on deep learning and best feature selection. Our major contributions of this work are as follows:

- Proposed an improved version of ResNet-50 CNN architecture with addition of three new residual blocks that reduced the parameters and improved the accuracy than the original ResNet-50.
- Automatically initialize the hyperparameters using Jaya Algorithm instead of using human knowledge.
- Designed a new Stacked Autoencoders network that consists of five convolutional layer in each—encoder and decoder. In addition, it is consists of 100 and 50 hidden layers.
- Proposed a parallel pooling layer fusion technique that fused the features of improved ResNet-50 model and Stacked Autoencoders.
- Proposed an improved version of optimization algorithm called improved Gray Wolf optimization.

## Literature review

Much research has been done in computer vision for classifying brain tumor modalities such as T1, T2, T1CE, and Flair. Each technique is based on several steps: tumor enhancement, feature extraction, and classification. Ginni et al.^24^ presented an ensemble-based brain tumor detection and Classification framework. In the presented framework, they considered the following challenges: boundary, shape, and volume, and named a few more. Initially, they used the OTSU thresholding method for tumor segmentation and later extracted SWT, PCA, and texture features for classification. They employed a hybrid ensemble method based on the majority voting scheme for improved classification. The experimental process of the presented framework was performed on privately collected 2552 images and obtained an improved accuracy of 97.2% than the recently introduced techniques. The suggested technique works well for only a small amount of images (dataset); hence, it is unsuitable for large datasets. In another work, Soumick et al.^25^ used a classification technique for brain tumor classification using a deep spatio-spatial model. The technique is influenced by spatiotemporal models used in the action recognition domain. They considered the 3D volumetric data of MRI images as spatiospatial. The variants of the ResNet model (i.e., ResNet (2 + 1)D and ResNet Mixed Convolution) were employed for this purpose. Comparative analysis with ResNet18 showed that variants performed better in accuracy (i.e., 96.98%) and computational time. They used the BraTS 2019 dataset. The proposed technique was employed only on one modality of MRI images (i.e., T1), but the remaining three (i.e., FLAIR, T2 & T1ce) were left unattended. Therefore, this technique is not suitable for all modalities of MRI images. Hapsari et al.^26^ introduced a performance enhancement technique for brain tumor classification using a convolutional neural network (CNN). They have improved the hyper-parameter tuning method for CNNs based on VGG16 and used an ADAM optimizer. They named their technique as enhanced CNN (en-CNN). They applied the proposed technique to the four modalities of MRI sequences (i.e., T1, T1CE, T2, and Flair) on the BRATS 2018 dataset and achieved an accuracy of 95.5% for T1, 95.5% for T1CE, 94% for T2 and 97% for FLAIR modality. They did not discuss any drawbacks of the technique. Still, Yang et al.^27^ highlighted many drawbacks of VGG, including computationally expensive for extensive data and gradient explosion problems that can occur during model training. A fusion-based technique for different modalities of MRI sequences (i.e., T1, T1CE, T2, and Flair) was put forward by Javaria et al.^28^, in which they had utilized Discrete Wavelet Transform (DWT) and Daubeches Wavelet Kernal (DWK) for fusion process that was aimed at fusing the structural and textual information. They utilized the global thresholding method and used a a convolutional neural network to classify the MRI sequence into tumor and no tumor area. The datasets used for the experimental process are BraTS2012, 2013, 2013 leader board, 2015, and 2018, and achieved 97%, 98%, 96%, 100%, and 97% accuracy, respectively.

In continuation of the classification techniques, Zahid et al.^29^ offered a technique in which they fine-tuned the ResNet101 model for the Classification of MRI sequences of different modalities. The model causes duplicate features, reducing accuracy and increased computational cost. They tackled the mentioned problem by using particle swarm optimization and differential evaluation to find the best features. Moreover, they fused the optimal features and applied the Principle Component Analysis (PCA) technique to filter out the best optimal features for classification. The final classification was done using a medium neural network. They achieved an accuracy of 96.7%. They have used a fusion technique that drastically enhanced the time complexity. In another work by Muhammad et al.^26^, they developed a novel technique for brain tumor segmentation that uses Particle Swarm Optimization (PSO) and a deep CNN model for classification to lessen false positives while classifying the segmented pictures into benign and malignant classes. They used the BraTS 2018 dataset for classification purposes. The segmentation results were 98.11% and 98.25%, while the classification results were 98.9% accurate. The drawback is that the PSO threshold value was selected manually in the suggested technique instead of some optimized automatic selection. Desouza et al.^30^ used radiomic properties and neural and ensemble learning-based methods to classify MRI sequences of different modalities (i.e., T1, T2, and Flair) into healthy glioma, meningioma, and pituitary classes. They experimented with K-fold cross-validation and showed that the eXtreme Gradient Boosting (XGBoost) framework outperforms the deep neural network after obtaining 88.51% accuracy. The deep neural network achieved an accuracy of 87.09%. The proposed technique does not include the T1CE modality of the MRI sequence. So, the suggested method cannot work on it. In their work, Gupta et al.^31^ offered a quick classification technique to diagnose brain malignancies. Using volumetric Fluid Attenuated Inversion Recovery (FLAIR) acquisition, data from 200 participants were acquired. They used 12 relevant slices to be utilized as the patient feature set for classification. Principal Component Analysis (PCA) and Discrete Wavelet Transform (DWT) were used to extract and select features. The effectiveness of different classifiers was examined, including Support Vector Machine (SVM), k-nearest Neighbor (k-NN), Classification and Regression Tree (CART), and Random Forest. K-fold cross-validation was applied to each train-test ratio. They found SVM as the best classifier and obtained 88% accuracy. However, the technique failed to classify tumors near the scalp and the brighter objects in the FLAIR sequence.

A multi-modal approach was suggested by Rajasree et al.^32^ for several modalities of magnetic resonance images (MRI), a Multiscale Multimodal Convolutional Neural Network (MSMCNN.) Along with a Long Short-Term Memory (LSTM) based deep learning semantic segmentation strategy is employed. The methodology assigned a class label to each pixel in the image. Using a multiscale U-NET-based deep convolution network, they employed multimodal convolutions divided into three different scale patches depending on a pixel level. The three paths were then merged in the LSTM network to determine the tumor classifications. They have applied this technique to the BraTS 15 dataset. They achieved an accuracy of 96% using their technique. The presented technique requires clinical validation, and the model requires a large dataset for training. Sharma et al.^33^ suggested a technique in which pre-trained Visual Geometry Group (VGG19) and Dense Convolutional Network (DenseNet201) models were used along with transfer learning-based techniques. Adam Optimizer was used for the optimization of features with both models. Data augmentation and normalization processes followed both of these models to improve the overall model accuracy. An accuracy of 98% was achieved with the VGG19 model, and an accuracy of 96% was attained using the DenseNet201 model. The dataset, consisting of 257 images, was collected. One hundred fifty-seven images contained brain tumors, and the rest were healthy. They used only axial data of brain tumor images; the technique was not used for coronal and sagittal datasets. Guo et al.^34^ developed a technique to improve the classification accuracy of the glioma subtype by using MRI modalities (i.e., T1, T2, T1ce, and Flair). To achieve this purpose, they used a fusion-based network. They used features extracted from segmentation as input to the DenseNet model during training. The predicted probabilities of the pre-trained models acquired in the training stage were combined during the inferring stage using a linear weighted module based on a decision fusion strategy. The proposed technique showed an accuracy of 87%. In their work, Fu et al.^35^ solved the problem of noise addition by a traditional fusion-based technique using Generative Adversarial Networks (GANs) that can ultimately affect accuracy. Another work proposed by Rehman et al.^36^ introduced a technique that can microscopically detect and classify brain tumors. They employed a 3-dimensional Convolutional Neural Network (3D-CNN) and feature selection architecture. Brain tumors are extracted using a 3D-CNN and then passed to a pre-trained CNN model for feature extraction. After the extracted features are transferred to the correlation-based selection technique, the best features are chosen as an output. Before the final classification, these selected features are validated using a feed-forward neural network. This technique used BraTS 2015, 2017 and 2018 datasets with an accuracy of 98.32%, 96.97%, and 92.67%. Their method achieved a considerable performance, but the classification time is prolonged compared to training the pre-trained model using original MRI scans.

## Methodology

**Figure 1:**
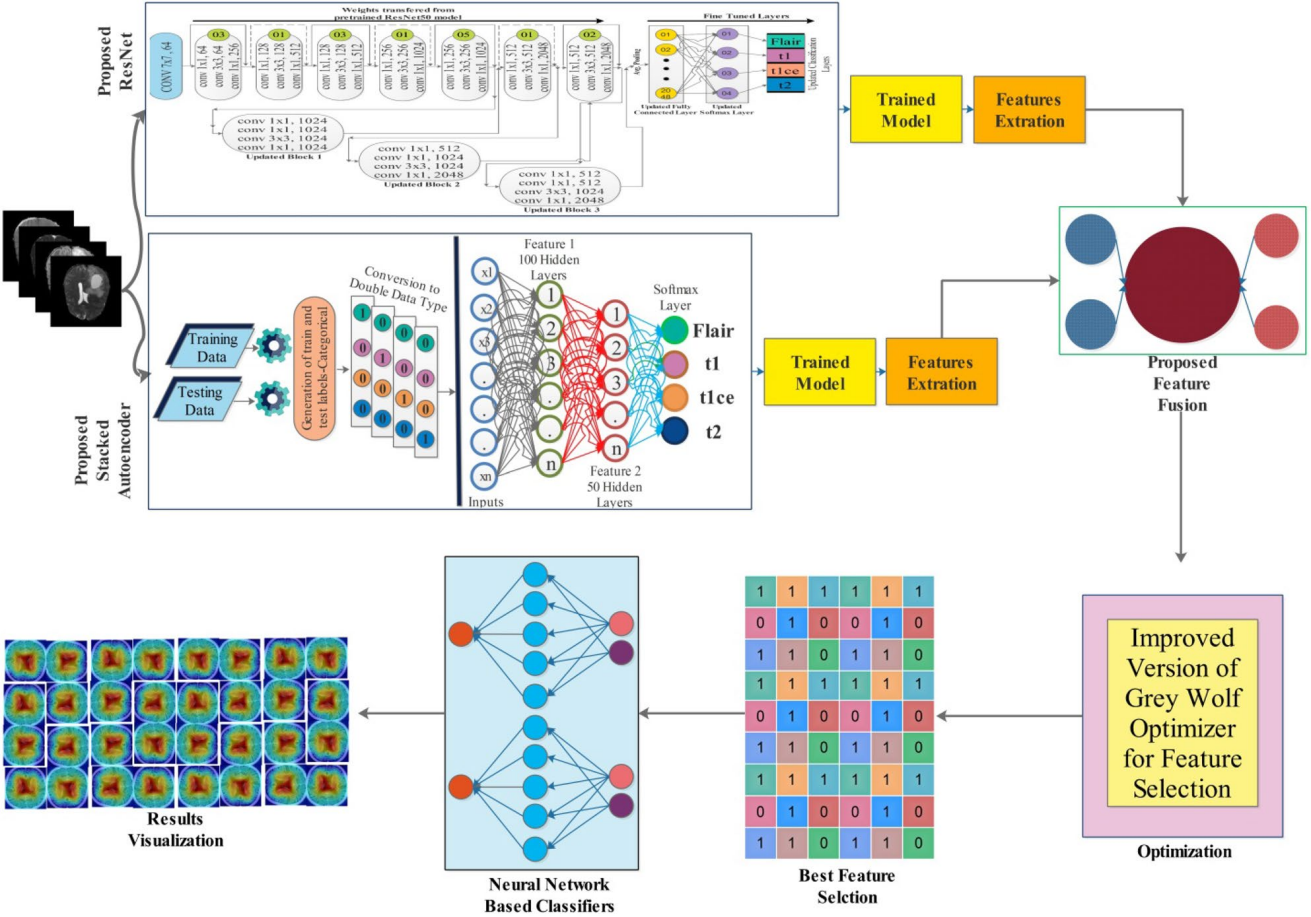
Proposed methodology of brain tumor classification using improved ResNet and Stack Auto-Encoder optimal features.

This work's proposed methodology is presented in this section using mathematical formulation and visual results. Figure 1 shows the proposed methodology for brain tumor visualization and classification. The experimental work started by obtaining publicly available MRI-based brain tumor datasets such as BraTS2020^37^ and BraTS2021^38^. After selecting datasets, a pre-trained model (ResNet50) is improved by adding a few layers and then employing a particle swarm optimization to select hyperparameters. After selecting hyperparameters, the improved network is trained from scratch, and obtained a new trained model that is further utilized for the feature extraction. In addition, this network is analyzed using a GradCAM-based visualization. Simultaneously, a Stack auto-encoder network is designed and trained on the selected datasets later utilized for the feature extraction. Features of both networks are extracted and fused using a serial-based minimum distance approach and further optimized by employing a Grey Wolf and Jaya hybrid optimization algorithm. In the last, selected features are classified using neural network classifiers for the final classification accuracy.

### Dataset

Two datasets have been selected for this work, BraTS2020 and BraTS2021 challenge. The first challenge was conducted in 2012. The subsequent challenges are now conducted every year by MICCAI^39^. The same dataset is used for other purposes^40,41^. Both datasets are divided into four MRI modalities: Flair, t1, t1ce, and t2.

The BraTS20 dataset contains 369 patients' data. Each file contains 155 slices in the image format. We have selected 75 (slices no. 40 to 114) slices for every patient from the middle portion of slices due to the enrichment of features that can be extracted. The lower end (slice numbers 1–39) and upper end (slice numbers 115–155) slices were not part of our experiment due to lesser information containment in the MRI scan. Each class of this dataset contains 27,675 images; hence, the total number of images is 110,700.

**Figure 2:**
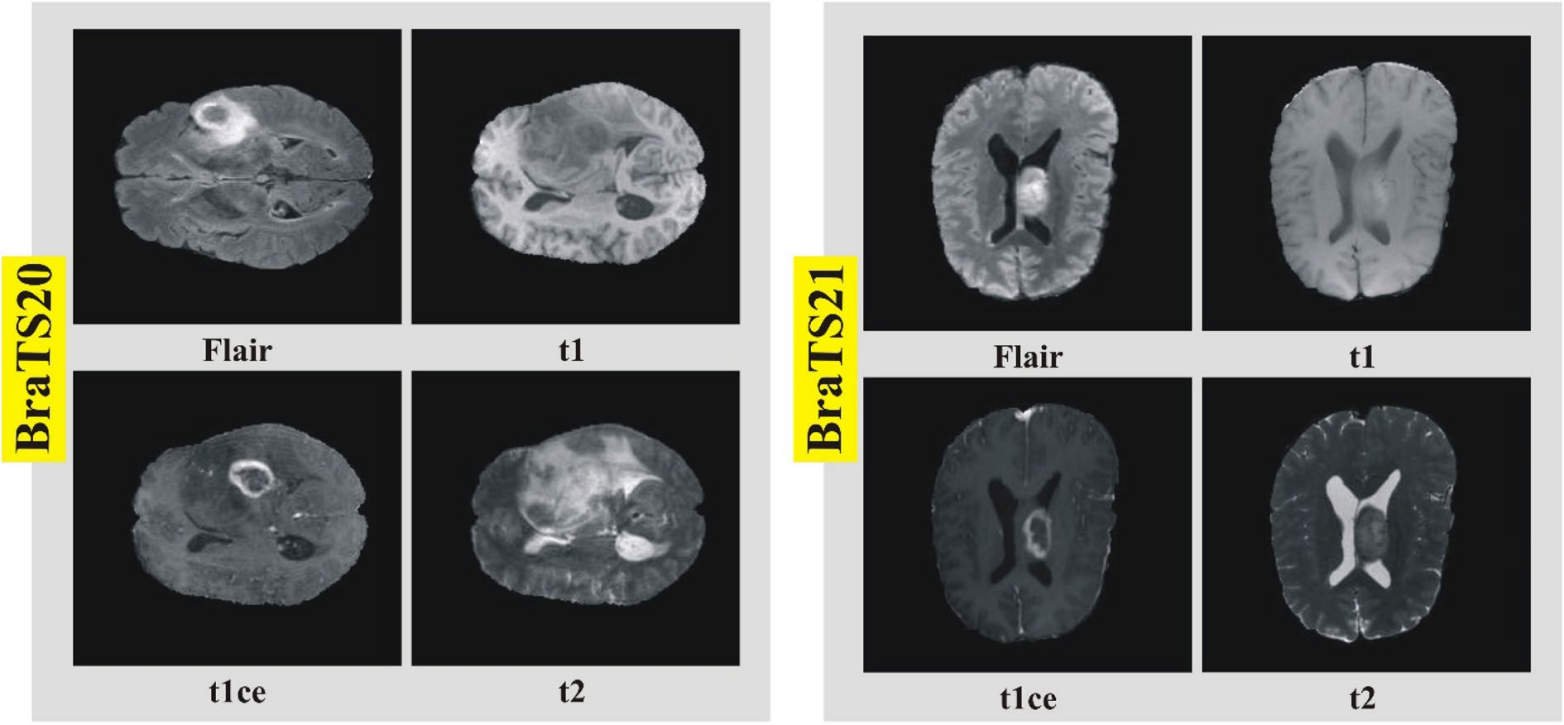
Sample MRI scans of brain tumor modalities for selected datasets.

The BraTS2021 dataset contains data from 1251 patients. The number of images in each class is 193,905, and the total is 775,620. To conduct our experiments, we took the data of 144 patients. The dataset is carefully selected from each patient that contains the complete tumor information. The number of images in each class was 6050, and the total number in all classes was 24,200. Figure 2 shows a sample MRI images of brain tumor modalities classification.

### Novelty 1: modified ResNet-50 pre-trained model

A pre-trained model is a machine learning model already trained on a sizable dataset and can be used as the basis for a new machine learning job. The resNet-50 model has been trained on the ImageNet dataset, a collection of over 14 million images categorized under 1000 distinct classes and many ontological subclasses^42^. Microsoft researchers unveiled the ResNet-50 convolutional neural network design in 2015^43^.

The ResNet-50 model has five stages, each with convolutional layers, batch normalization, and activation functions. The first step includes a 7 × 7 convolutional layer with a stride of 2 followed by a max pooling layer. The feature maps' spatial dimensions are halved at the end of each stage, and the following stages have multiple convolutional layer blocks with residual connections. The model has a global average pooling layer and a fully connected layer with softmax activation for classification. The use of residual connections is a significant characteristic of ResNet-50, helping the network learn more effectively by preventing vanishing gradients.

The modified ResNet-50 model accepts a size of 224 × 224 × 3 as an input layer. Large datasets need more computation power. So, more layers are added to extract the best features from the data. Introducing new layers in CNN usually comprises the problem of vanishing gradient while updating the weights during backpropagation. Backpropagation is mathematically defined as follows:

1 $${\delta }^{L}={\Sigma }^{\prime}\left({Z}^{L}\right){\nabla }_{a}C$$

2 $${\delta }^{{^{\prime}}}= {\Sigma }^{{^{\prime}}}({Z}^{l})({\omega }^{l+1}{)}^{T}{\delta }^{l+1}$$

3 $${\delta }{\prime}= {\Sigma }{\prime}({Z}^{l})({\omega }^{l+1}{)}^{T}{\dots \Sigma }{\prime}({Z}^{L-1})({\omega }^{L}{)}^{T}{\Sigma }{\prime}\left({Z}^{L}\right){\nabla }_{a}C$$

**Figure 3:**
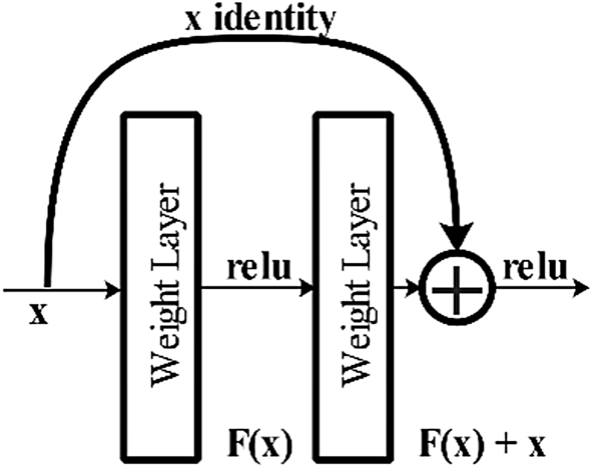
ResNet-50 skip connection.

Backpropagation is considered a greedy approach for an optimization problem. The greedy approach works well with local optimization but not with global optimization. As in the BP, the traverse down is performed for the network; hence, these small derivatives are multiplied together, leading to a rapid decline in the gradient that becomes progressively smaller as we near the initial layers. Therefore, a vanishing gradient problem occurs. ResNet employs an alternative shortcut path to address the vanishing gradient issue to allow for gradient flow. The model uses an identity mapping technique, allowing it to bypass a convolutional neural network weight layer when unnecessary. This approach aids in avoiding overfitting to the training set^44^. Visually, it is shown in Fig. 3. The skip connection in residual networks enables overcoming the vanishing gradients in ResNet-50 model.

**Figure 4:**
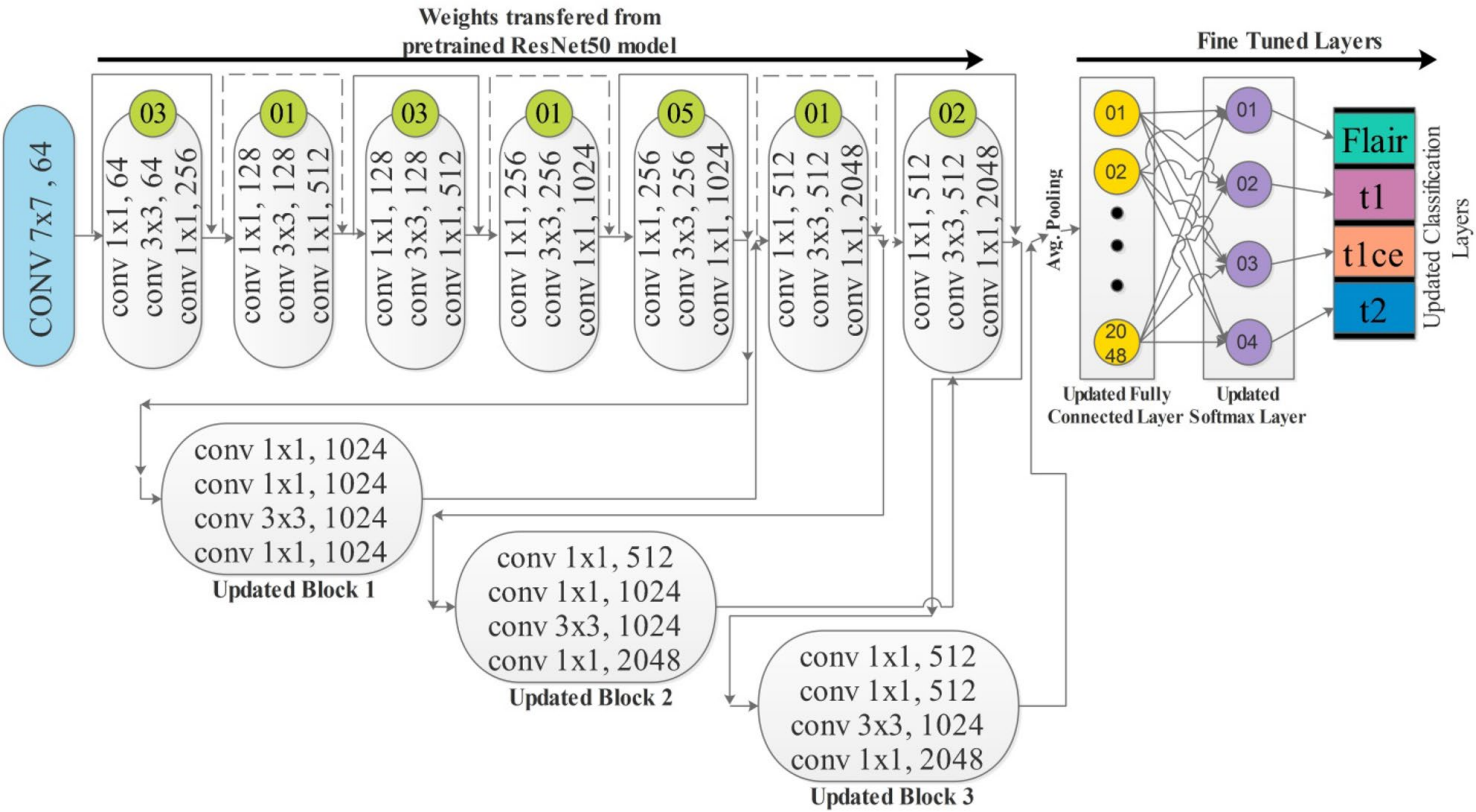
Proposed improved version of ResNet-50 CNN architecture.

*Modification and Training* In the first modification step, we added three new residual blocks, consisting of 4 convolutional layers. Each convolutional layer has $1\times 1$ and $3\times 3$ convolutional filters, and the stride is $1$. After the first block, one convolutional layer of filter size 3 × 3 and stride 1 is added. The ReLu activation layer is added after each convolutional layer to resolve the nonlinearity issue. After adding three blocks, a global average pooling layer is added, as show in Fig. 4. In the end, a fully connected layer, softmax layer, and classification layers have been added and trained on the model on the selected dataset. The training process is conducted through transfer learning^45^ without freezing any layer. The parameters of the new fully connected layer and overall parameters are defined in the Table 1 below. The hyperparameters have been selected based on the Jaya optimization algorithm^46^. The purpose of this step is to fully automate the system for the initialization of hyperparameters without involving human experience.

**Table 1:** Parameters of trained modified ResNet-50 CNN architecture.

Layer name	Fully connected layer
WeightLearnRateFactor	10
BiasLearnRateFactor	10
Optimizer	Stochastic gradient descent momentum (sgdm)
Maximum number of epochs	100
Minimum batch size	256
Initial learner rate	0.001

### Novelty 2: stack auto-encoder network

A Stacked Auto-Encoder is a deep learning neural network architecture comprising multiple levels of encoders, which is succeeded by several layers of decoders^47^. The primary objective of this architecture is to learn a compressed representation of the input data that can be utilized for tasks like dimensionality reduction or representation learning. The Encoder component of the network transforms the input data into a bottleneck layer or latent representation, representing the data in lower dimensions. The decoder component then maps the lower-dimensional representation to the original input data space. The network is trained to minimize the difference between the decoder output and the original input data, ensuring the decoder output is as close to the input data. The loss function is used to minimize the difference.

An Auto-encoder with several layers $(N)$, and the input data with $(D)$ dimensions, then encoder, decoder, and loss function can be calculated using the below equations.

*Encoder* Consider each layer, the encoding function can be defined as:

4 $${en}_{i}={act}_{i}\left({wt}_{i}*{op}_{i}-1+{bias}_{i}\right)$$

where output ${en}_{i}$ is encoding function, ${act}_{i}$ is the activation function, ${wt}_{i}$ is the weight matrix, ${op}_{i}-1$ is an output of the previous layer, and ${bias}_{i}$ is the bias vector of layer $i$.

*Decoding* The decoding function of layer $i$ can be defined as:

5 $${de}_{i}={dact}_{i}\left({dwt}_{i}*{den}_{i}+{dbias}_{i}\right)$$

where ${de}_{i}$ is the output of the decoding function, ${dact}_{i}$ activation function, ${den}_{i}$ is the input from previous encoding layer, ${dwt}_{i}$ is weight matrix and ${dbias}_{i}$ is the bias vector, of decoding layer $i$.

*Loss function* The mean squared error between the input data and the output of the terminating decoding layer is acts as a loss function and can be denoted with following equation:

6 $$L=\frac{1}{\left(2N\right)}*\Vert {\text{I}}-{\text{dOUT}}\Vert ^2$$

**Figure 5:**
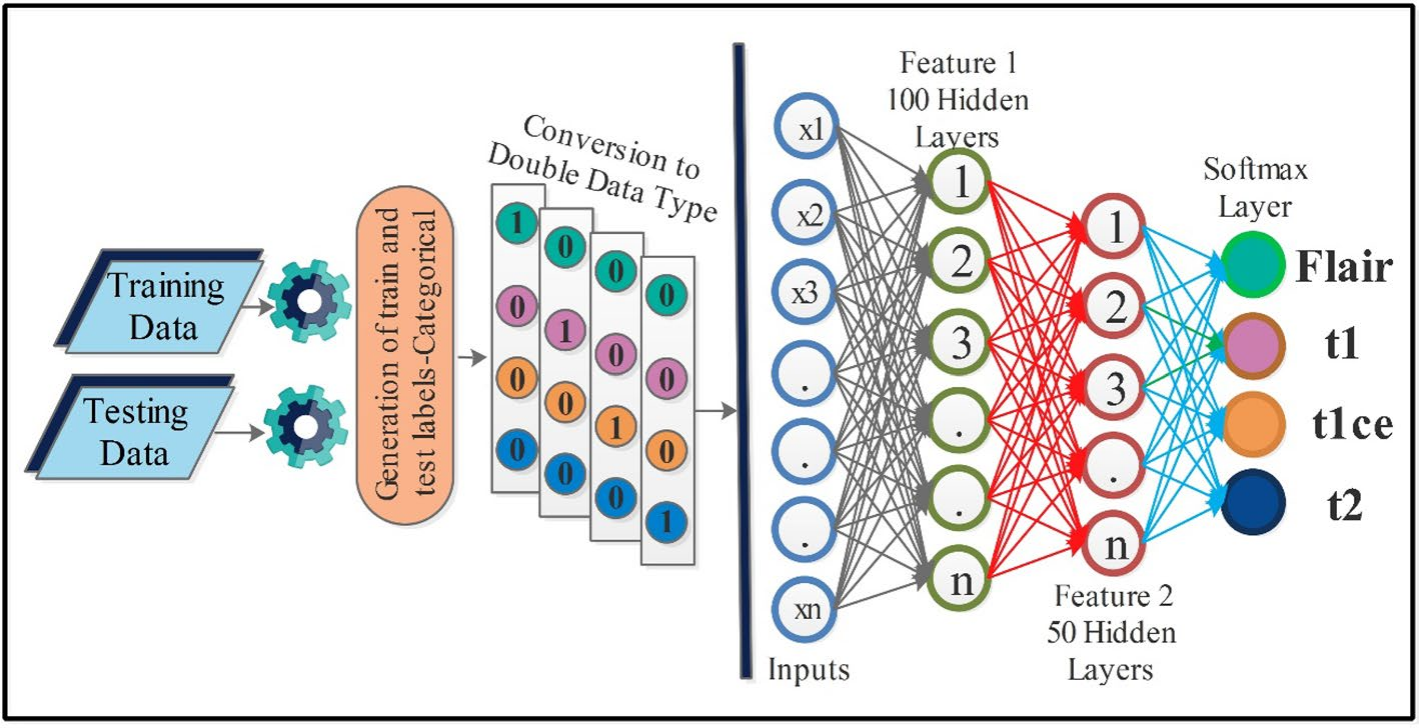
Proposed Stack Autoencoder network for features extraction.

In the equation, $I$ denotes the input, ${\text{dOUT}}$ is the output of the terminating layer, and the squared Euclidean norm is represented by ǁ·ǁ^2^. After the assignment of these functions, training is performed, and a trained model is later utilized for the feature extraction process. An overall architecture of proposed Stacked Autoencoders is showing in Fig. 5.

### Feature extraction

After training two deep learning models named Modified ResNet-50 and Stack Auto Encoder–Decoder, features are extracted from the deeper layer. In the Modified ResNet-50 architecture, the global average pooling layer is employed for the feature extraction, whereas the last decoder convolutional layer is opted for the encoder-decoder network feature extraction. Both feature vectors are defined as follows:

7 $${\psi }_{fv1}=\varrho \left({\vartheta }_{k}, gap\right)$$

8 $${\psi }_{fv2}=\Upsilon \left({\vartheta }_{k}, Dconv\right)$$

where, ${\vartheta }_{k}$ denotes the data samples that are utilized for the feature extraction, ${\psi }_{fv1}$ denotes the Modified Resnet-50 architecture, ${\psi }_{fv2}$ denotes the Stack Auto Encoder-Decoder network, and $Dconv$ is the decoder convolutional layer. The size of both feature vectors is $N\times 1024$ and $N\times 1236$, respectively.

### Novelty 3: proposed feature fusion and selection

Feature fusion is the process of combining information of more than 1 sources into a single source for the improved information^48^. In this work, a novel parallel pooling concept-based approach is proposed for the fusion of both feature vectors. Consider we have two feature vectors ${\psi }_{fv1}$ and ${\psi }_{fv2}$ of dimensions $N\times 1024$ and $N\times 1236$. As per the parallel fusion definition, both vectors should have an equal length; therefore, we performed mean padding ${\psi }_{fv1}$. After that, a pooling operation is performed of filter size $2\times 2$ and stride 1.

9 $$Fusion(k1)=\widetilde{\Delta }\left({\psi }_{fv1}, stride, filter\right)$$

10 $$Fusion(k2)=\widetilde{\Delta }\left({\psi }_{fv2}, stride, filter\right)$$

11 $$Fusion(\widetilde{k})=\left(\begin{array}{c}Fusion\left(k1\right)\\ Fusion(k2)\end{array}\right)$$

where $Fusion(k1)$ and $Fusion(k2)$ denote the resultant pooling vectors of dimensional 512 and 618, after that, both vectors are combined using Eq. (11) and obtained feature vectors of dimensional $N\times 1130$. The fused vector is optimized using an improved version of Grey Wolf Optimization algorithm with update criteria of Jaya algorithm.

Feature selection is selecting the best features later utilized for the classification^49^. This step aims to improve the accuracy and reduce the computational cost. The original extracted features consist of redundant and irrelevant information impacting classification accuracy and computational time. In addition, cleaning irrelevant and redundant information is important to obtain the classification results quickly. To address these challenges, a hybrid technique is proposed based on the initial selection of Grey Wolf Optimization and later again selected through an update equation.

The GWO is a nature-inspired algorithm presented by Mirjalili et al.^50^ that impersonates grey wolves' hunting (optimized) behavior. Consider we have two feature vectors. ${\psi }_{fv1}$ and ${\psi }_{fv2}$ of dimensions $N\times 1024$ and $N\times 1236$. We initialize the number of populations 50 and iterations 200. The GWO is work in the following five steps:

*Social hierarchy* The hunting or dominance hierarchy involves alpha $(\alpha$), beta $(\beta ),$ and delta $(\delta )$. The omega $(\omega )$ member follows the dominance of all.

*Encircling prey* To hunt, grey wolves encircle the prey, the encircling behavior can be modeled mathematically as follows:

12 $$\overrightarrow{L}= \overrightarrow{B}.{\overrightarrow{Z}}_{p}\left(t\right)- \overrightarrow{Z}\left(t\right)$$

13 $$\overrightarrow{Z}\left(t+1\right)= {\overrightarrow{Z}}_{p}\left(t\right)- \overrightarrow{M}.\overrightarrow{L}$$

where current iteration is indicated by $m$. The coefficient vectors are denoted by $\overrightarrow{M}$ and $\overrightarrow{B}$. The position vector of the prey is ${\overrightarrow{Z}}_{p}$. The position vector of the prey is indicated by $\overrightarrow{Z}$. The coefficient vectors are denoted by $\overrightarrow{M}$ and $\overrightarrow{B}$ can be calculated as follows:

14 $$\overrightarrow{M}= 2\overrightarrow{g}.\overrightarrow{{h}_{1}}- \overrightarrow{g}$$

15 $$\overrightarrow{M}= 2.\overrightarrow{{h}_{2}}$$

An iteration-based linear decrement is done from $2$ to $0$ in the components of $\overrightarrow{g}$. The $\overrightarrow{{h}_{1}}$ and $\overrightarrow{{h}_{2}}$ are two vectors with the range of $[\mathrm{0,1}]$.

*Hunting* Grey wolves hunt their prey precisely, and it is usually guided by alpha $(\alpha$). The participation of beta $(\beta )$ and delta $(\delta )$ is occasion-based. In a real scenario, the location of the prey always changes. In order to mathematically represent the problem space. We assume that the location of the prey is known by alpha (the Best Solution), beta, and delta. So, the first three best solutions obtained so far are saved and on the basis of this, we oblige the other search agents to update their position. Mathematical notation is given as follows: -

16 $$\overrightarrow{{L}_{\alpha }}= \overrightarrow{{B}_{1}}.{\overrightarrow{Z}}_{\alpha }- \overrightarrow{Z}, \overrightarrow{{L}_{\beta }}= \overrightarrow{{B}_{2}}.{\overrightarrow{Z}}_{\beta }- \overrightarrow{Z},\overrightarrow{{L}_{\delta }}= \overrightarrow{{B}_{3}}.{\overrightarrow{Z}}_{\delta }- \overrightarrow{Z}$$

17 $${\overrightarrow{Z}}_{1}= {\overrightarrow{Z}}_{\alpha }-{\overrightarrow{M}}_{1}.\left({\overrightarrow{L}}_{\alpha }\right), {\overrightarrow{Z}}_{2}= {\overrightarrow{Z}}_{\beta }-{\overrightarrow{M}}_{2}.\left({\overrightarrow{L}}_{\beta }\right), {\overrightarrow{Z}}_{3}= {\overrightarrow{Z}}_{\delta }-{\overrightarrow{M}}_{3}.({\overrightarrow{L}}_{\delta })$$

18 $$\overrightarrow{Z}\left(m+1\right)= \frac{{\overrightarrow{Z}}_{1}+{\overrightarrow{Z}}_{2}+{\overrightarrow{Z}}_{3}}{3}$$

*Attacking prey* As the prey stops moving, the value of $\overrightarrow{g}$ decreases. The variability range of $\overrightarrow{M}$ is also decreased by $\overrightarrow{g}$. In other words, we can say that vector $\overrightarrow{M}$ has an interval $[-2g,2g],$ and the value of $g$ iteratively decreased from $2$ to $0$. When Randomized values of vector $\overrightarrow{M}$ reaches between the interval $[-\mathrm{1,1}]$, the new position of the searching agent can be any between the current position and the prey's position. In this position, the wolves usually chose to attack the prey.

*Search for prey* To search for prey, grey wolves diverge and converge to attack the prey. Searching for prey usually involves alpha, beta, and delta members. The divergence mechanism can be mathematically modeled by selecting the random values of the vector $\overrightarrow{M}$ greater than $1$ or less than − $1$. The grey wolf algorithm searches globally by keeping in view the divergence mechanism. The vector $\overrightarrow{B}$ can also help in divergence. It has a range of $[0,2]$ random values. It emphasizes avoiding the problem of local optima and stagnation during the final iterations of the exploration stage.

The fitness is computed in each iteration and the best solution. The extreme learning machine (ELM)^51^ classifier is employed as a fitness function in this work that computes the accuracy of each solution after each iteration. After all iterations, the best-selected solution is further optimized using an update function of Jaya optimization^52^. Mathematically, it is defined as follows:

19 $${X{\prime}}_{l,m,j}= {X}_{l,m,j}+{r}_{1,l,j}\left({X}_{l,best,j}-\left|{X}_{l,m,j}\right|\right)- {r}_{2,l,j}({X}_{l,worst,j}-\left|{X}_{l,m,j}\right|)$$

In the equation ${X}_{l,best,j}$ is the value of the variable $l$ for the best solution candidate, and ${X}_{l,worst,j}$ is the value of variable $l$ for the worst solution candidate. The variables ${r}_{1,l,j}$ and ${r}_{2,l,j}$ are random variables that are generated between $[\mathrm{0,1}]$ range for ${l}^{th}$ variable and in the ${j}^{th}$ iteration. The equation $+{r}_{1,l,j}\left({X}_{l,best,j}-\left|{X}_{l,m,j}\right|\right)$ moves the overall solution closer to the best solution, while the equation $- {r}_{2,l,j}({X}_{l,worst,j}-\left|{X}_{l,m,j}\right|)$ avoids the solution to be closer to the worst solution. The resultant value ${X{\prime}}_{l,m,j}$ is accepted if it provides us with better function values. The accepted resultant values are then saved in some variable and become the next iteration's input. In this paper, 100 iterations were selected for this second phase. The fitness value is computed in each iteration and the best solution ${\psi }_{fv1}^{sl}$ is selected after all iterations. The best selected solution is finally passed to neural networks and machine learning algorithms. A brief working of the entire proposed method is described under Algorithm 1.
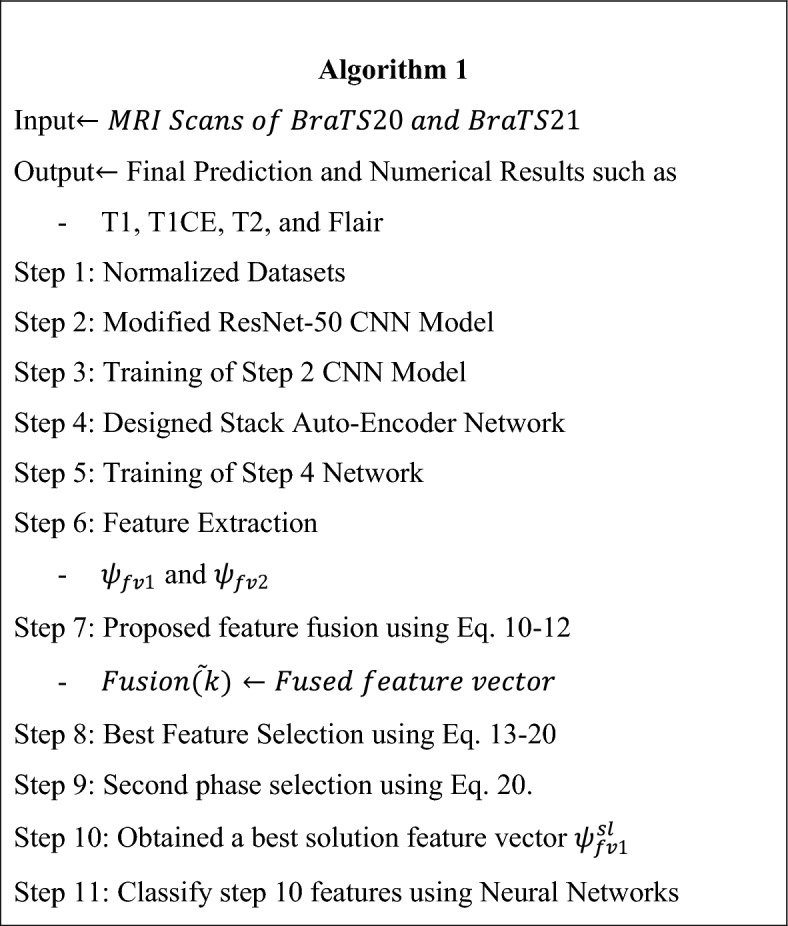


## Proposed results and discussion

### Experimental setup

To perform experiments using improved version of ResNet-50, we have selected stochastic gradient descent with momentum (SGDM) to accelerate the gradient vectors for faster convergence at convolutional layers. We have used the k-fold cross-validation and selected the value of k = 10 for the model's training accuracy. We have divided the whole dataset into training and testing by the ratio of 50:50. During the algorithms learning process, we select a subset called a mini batch size and set its value to 256. Ultimately, we set the initial learner rate equal to 0.0001. On the other hand, while performing experiments with a Stacked Autoencoder, we have used two stacks of autoencoders. In the first stack, we selected hidden size = 100, the maximum number of epochs to 5, L2WeightRegularization = 0.004, SparsityRegularization = 4, and SparsityProportion = 0.15. In the second stack, we have used selected hidden size = 50, max number of epochs to 5, L2WeightRegularization = 0.002, SparsityRegularization = four, and SparsityProportion = 0.1. Moreover, the experiments were performed on MATLAB R2022a on a computer with 16 GB of RAM and a 12 GB Graphics Processing Unit (GPU) of RTX3060.

### Datasets and performance measures

We have used MICCAI BraTS 2020 and MICCAI BraTS 2021 brain tumor MRI datasets for our experiments. Both of these datasets are available publically for research purposes. We use different performance measures to evaluate our methodology's experimental results on these datasets. Performance measures in machine learning are metrics used to evaluate how well a model or algorithm can predict outcomes based on the available data. The selected measures are sensitivity rate, false negative rate (FNR), precision rate, and area under the Curve of each classifier. Additionally, we have recorded the accuracy and training time for each classifier. More information regarding these performance protocols can be found in Table 2.

**Table 2:** Performance measures used to validate the proposed methodology.

Name	Accuracy (%)	Time	Sensitivity rate (%)	False negative rate (%)	Precision rate (%)	Area under curve
Performance measure	$\frac{TP+TN}{TP+TN+FP+FN}$	*Seconds*	$\frac{TP}{TP+FP}$	$\frac{FN}{TP+FP}$	$\frac{TP}{TP+FN}$	$\underset{a}{\overset{b}{\int }}f\left(x\right)dx$

Where *TP* True Positive, *TN* True Negative, *FP* False Positive, *FN* False Negative.

### MICCAI BraTS 2020 results

**Figure 6:**
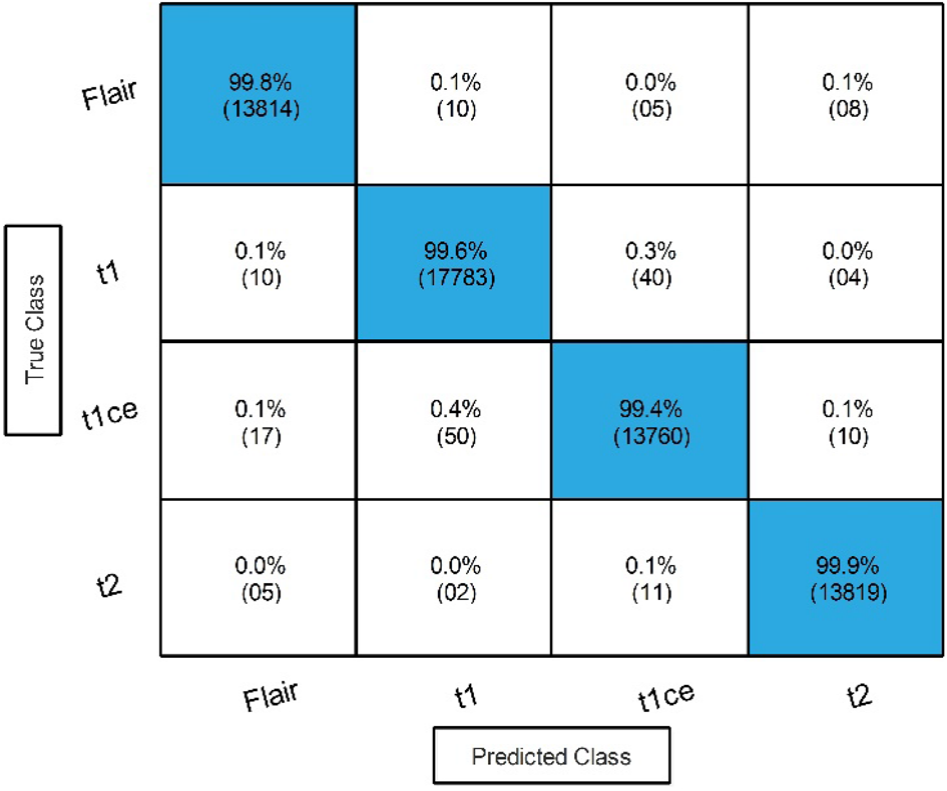
Confusion matrix of Tri-layered Neural Network for an improved version of ResNet-50 CNN architecture.

Results of the proposed framework have been computed in several steps to show the importance of each middle step. The improved ResNet-50 CNN architecture is selected in the first step, and classification is performed in the feature extraction phase. The results of this phase are given in Table 3. This table shows 99.70% best accuracy with a minimum noted time of 206.75 (s) for Medium Neural Networks. Figure 6 shows the confusion matrix of this step to verify the obtained performance measures. The diagonal values in this figure illustrated each modality class's correct prediction (number of true observation and true positive rate).

**Table 3:** Classification results of the improved version of ResNet-50 CNN architecture.

Classifier	Accuracy (%)	Time (s)	Sensitivity rate (%)	False negative rate (%)	Precision rate (%)	Area under curve (%)
WNN	**99.70**	277.30	**99.68**	**0.32**	**99.70**	**1.00**
Medium NN	99.60	236.82	99.70	0.30	99.70	1.00
Bi-LayerNN	99.64	358.60	99.68	0.32	99.65	1.00
Tri-Layered NN	99.58	**206.75**	99.68	0.32	99.68	1.00
Narrow NN	99.60	233.20	99.65	0.35	99.65	1.00

Significant values are in bold.

**Figure 7:**
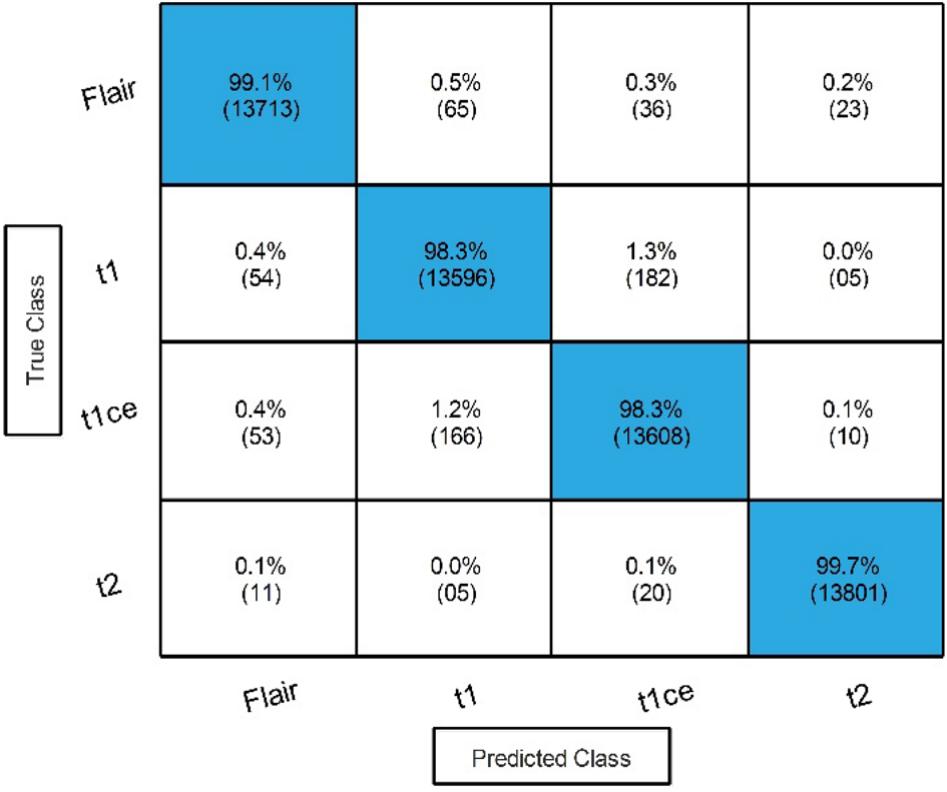
Confusion matrix of proposed stacked autoencoders.

In the second step, we employed Stacked Autoencoders architecture and extracted features, as shown in Fig. 4. The results of this step are given in Table 4. In this table, 98.90% best accuracy is noted for Wide Neural Network with 98.85% sensitivity rate and 98.85% precision rate. The other classifiers, named Medium Neural Network, Narrow Neural Network, and Tri-Layered Neural Network, obtained an accuracy of 97.90%, 92.70%, and 92.60%, respectively. The minimum accuracy was 92.40%, which was obtained by the Bi-Layered Neural Network classifier. Whereas the minimum noted time is 209.20 (sec) for Tri-Layered Neural Network. Figure 7 shows the confusion matrix for this step to verify the obtained performance measures. The diagonal values in this figure illustrate the correct prediction.

**Table 4:** Classification results by proposed Stacked Autoencoders model.

Classifier	Accuracy (%)	Time (s)	Sensitivity rate (%)	False negative rate (%)	Precision rate (%)	Area under curve (%)
WNN	**98.90**	303.70	**98.85**	**1.15**	**98.85**	**1.00**
Medium NN	97.90	232.83	97.90	2.10	97.88	1.00
Bi-LayerNN	92.70	223.5	92.75	7.25	92.77	0.985
Tri-Layered NN	92.60	237.30	92.60	7.40	92.63	0.988
Narrow NN	92.40	**209.20**	92.40	7.60	92.43	0.985

Significant values are in bold.

**Figure 8:**
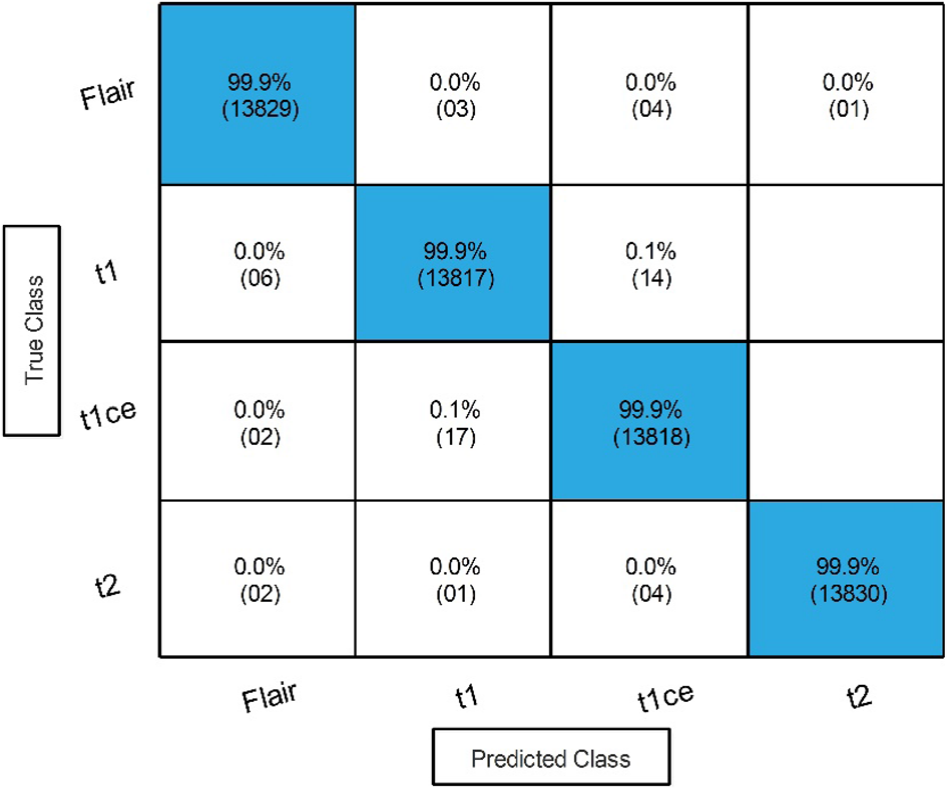
Confusion matrix of wide neural network using proposed fusion technique.

To enhance the accuracy, a novel fusion technique is utilized to fuse the information of both proposed models. The results of this step are given in Table 5. In this table, 99.90% best accuracy is noted for Wide Neural Network with 99.90% sensitivity rate and 99.80% precision rate. Whereas the minimum noted time is 249.38 (s) for Bi-Layered Neural Network. Figure 8 shows the confusion matrix for this step to verify the obtained performance measures. It is noted that after this step, the accuracy is improved by 0.2% and 1% for the improved ResNet-50 and proposed Stacked Autoencoders model, respectively. However, an increase occurs in the computational time after the fusion process. An optimization technique is proposed in the next step to address this challenge.

**Table 5:** Classification results after the fusion of improved ResNet-50 and proposed Stacked Autoencoder features.

Classifier	Accuracy (%)	Time (s)	Sensitivity rate (%)	False negative rate (%)	Precision rate (%)	Area under curve (%)
WNN	**99.90**	**338.42**	**99.90**	**0.10**	**99.80**	**1.00**
Medium NN	99.82	**303.59**	99.88	0.12	99.80	1.00
Bi-LayerNN	99.80	389.38	99.88	0.12	99.80	1.00
Tri-Layered NN	99.82	**304.8**	99.88	0.12	99.80	1.00
Narrow NN	99.78	**363.24**	99.80	0.12	99.80	1.00

Significant values are in bold.

**Figure 9:**
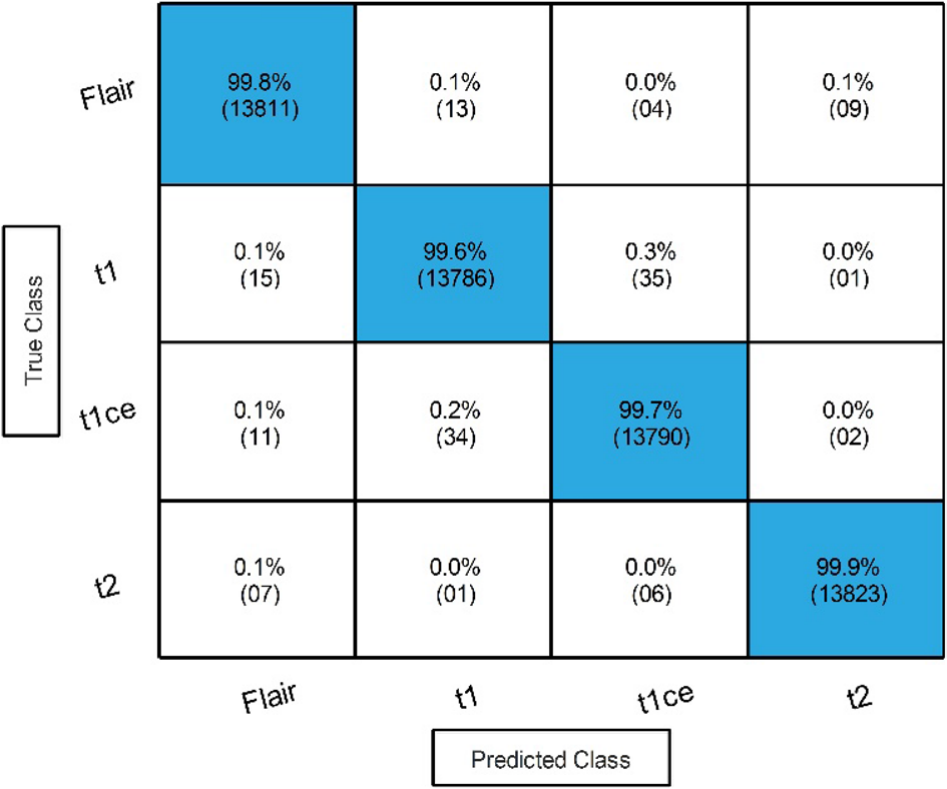
Confusion matrix of wide neural network using proposed optimization algorithm.

**Figure 10:**
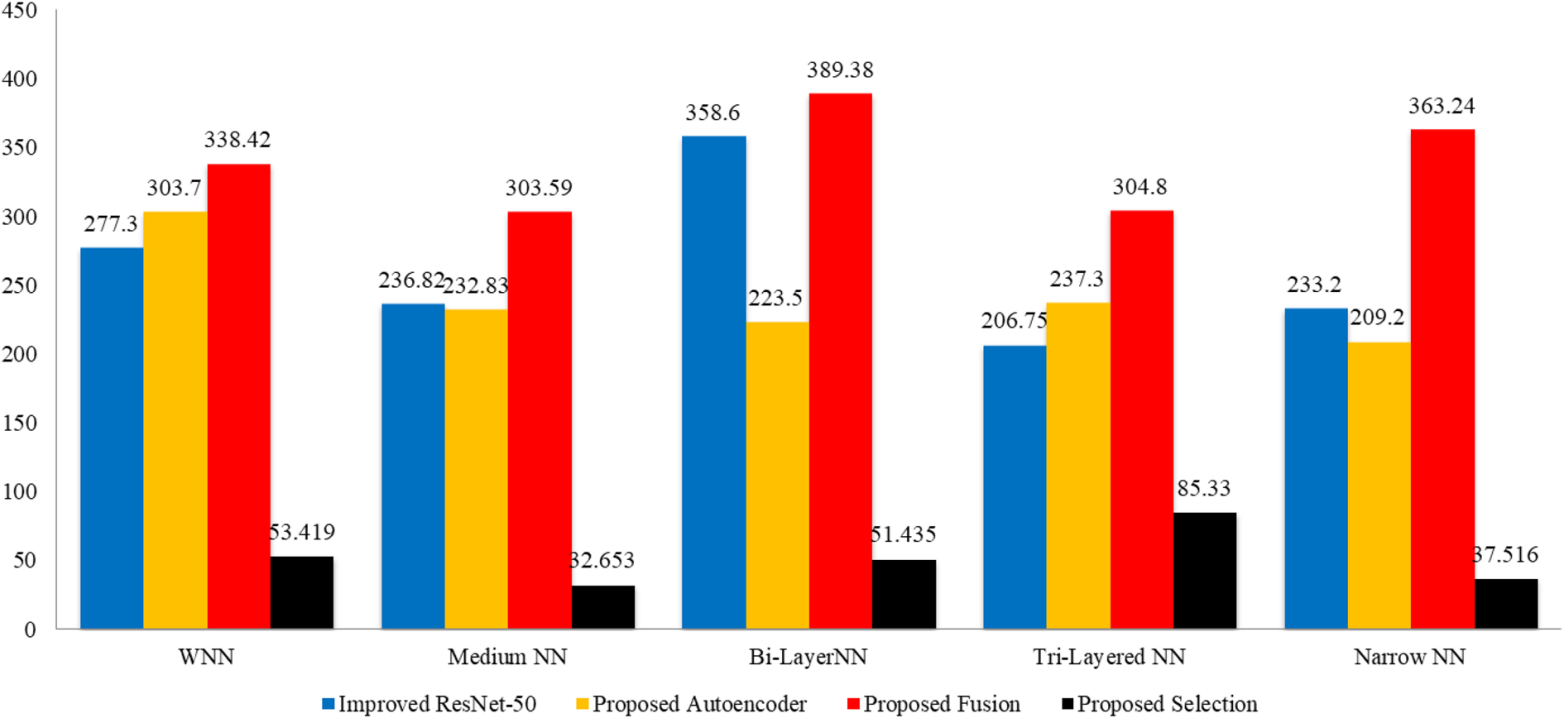
Computational time based comparison among all middle steps for BraTS2020 dataset.

In the final step, the proposed optimization algorithm is applied, and the best features are selected. The results of the proposed optimization step are given in Table 6. In this table, 99.80% best accuracy is noted for WNN, with a 99.75% sensitivity rate and 99.78% precision rate. The MNN obtained the second-highest accuracy of 99.70%, while the Bi-Layered Neural Network, Tri-Layered NN, and Narrow NN classifier obtained the minimum accuracy of 99.60%. Whereas the minimum noted time is 32.653 (sec) for Medium Neural Networks. Figure 9 shows the confusion matrix for this step to verify the obtained performance measures. This final step has improved the time complexity many folds than that of previous steps. The time complexity is decreased to approximately 87% in the third step, 98% in the second step, and approximately 95% in the first step. There is a negligible decrease (i.e., 0.1%) in accuracy in the third step due to dropping a few features during the selection step. However, this minor decrease is tolerable by keeping in view the improvement in the computational time. Figure 10 shows a detailed visual comparison in terms of time among all middle steps. In this figure, it is clearly observed that the time is significantly reduced after employing the proposed optimization technique.

**Table 6:** Classification results of the proposed optimization technique using BraTS2020.

Classifier	Accuracy (%)	Time (s)	Sensitivity rate (%)	False negative rate (%)	Precision rate (%)	Area under curve (%)
WNN	**99.80**	53.419	**99.75**	**0.25**	**99.78**	**1.00**
Medium NN	99.70	**32.653**	99.70	0.30	99.70	1.00
Bi-LayerNN	99.60	51.435	99.63	0.37	99.63	1.00
Tri-Layered NN	99.60	85.33	99.63	0.37	99.63	1.00
Narrow NN	99.60	37.516	99.60	0.40	99.63	1.00

Significant values are in bold.

### MICCAI BraTS 2021 results

The same procedure of experiments was also carried out on the second dataset. The first step is performed for an improved version of ResNet-50, as shown in Fig. 4. Features are extracted from the global average pooling layer, and classification results are described in Table 7. The result describes that 99.90% best accuracy is noted. In addition, the minimum noted time is 41.692 (s) for Medium NN.

**Table 7:** Proposed classification results of improved ResNet-50 model on BraTS2021.

Classifier	Accuracy (%)	Time (s)	Sensitivity rate (%)	False negative rate (%)	Precision rate (%)	Area under curve (%)
Medium NN	**99.90**	**41.692**	**99.87**	**0.12**	**99.87**	**1.00**
Narrow NN	99.90	46.518	99.87	0.13	99.87	1.00
Wide NN	99.90	60.325	99.87	0.13	99.85	1.00
Bi-Layered NN	99.80	45.644	99.83	0.17	99.85	1.00
Tri-Layered NN	99.80	62.655	99.78	0.22	99.80	1.00

Significant values are in bold.

In the second experiment, the proposed Stacked Autoencoders model is utilized, and features are extracted. The results of this step are described in Table 8. The result describes that 99.60% best accuracy is noted along with 99.58% sensitivity rate and 99.55% precision rate for the Wide Neural Network classifier. Moreover, an accuracy of 99.50%, 98.80%, 98.80%, and 98.60% was noted for Medium, Bi-Layered, Narrow, and Tri-Layered Neural Networks, respectively. In addition, the minimum noted time is 67.558 (sec) for Medium NN. Compared to the previous experiment, the results show that the proposed Stacked Autoencoders obtained results in higher time (> 62%) than improved ResNet-50.

**Table 8:** Proposed classification results of stacked autoencoders model on BraTs2021 dataset.

Classifier	Accuracy (%)	Time (s)	Sensitivity rate (%)	False negative rate (%)	Precision rate (%)	Area under curve (%)
Wide NN	**99.60**	88.419	**99.58**	**0.42**	**99.55**	**1.00**
Medium NN	99.50	**67.558**	99.48	0.52	99.50	1.00
Bi-Layered NN	98.80	129.64	98.80	1.20	98.83	1.00
Narrow NN	98.80	217.99	98.80	1.20	98.75	1.00
Tri-Layered NN	98.60	208.53	98.55	1.45	98.55	1.00

Significant values are in bold.

**Figure 11:**
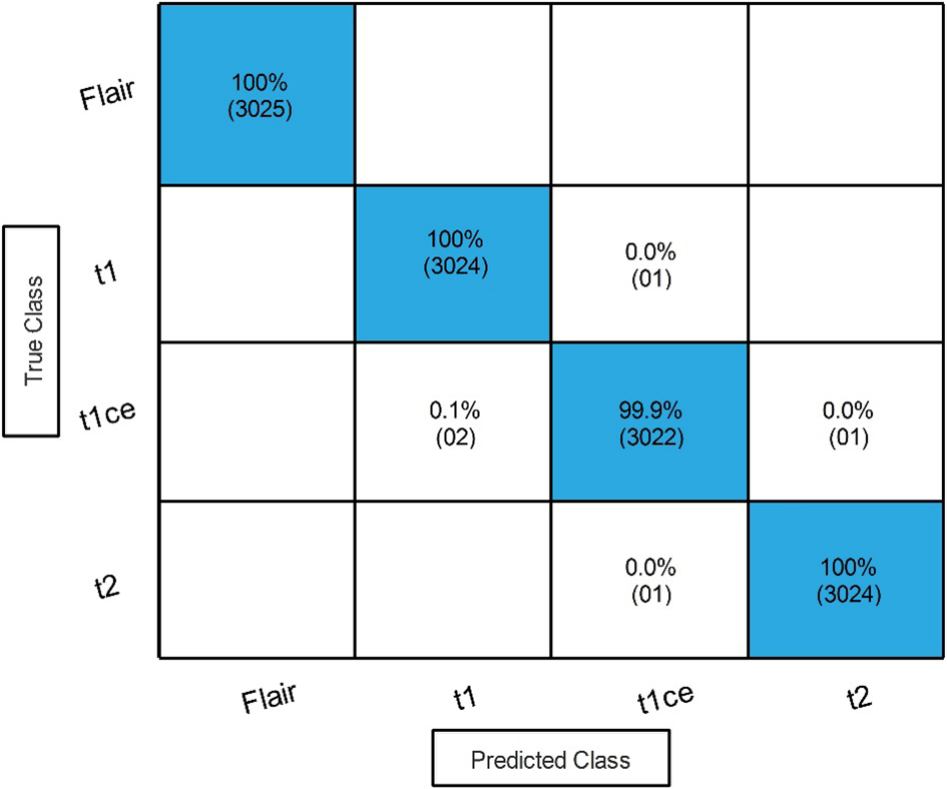
Confusion matrix of the best accuracy after employing the proposed fusion technique.

In the third step, a novel fusion technique is employed, and results are described in Table 9. The result describes that 100% best accuracy is noted along with a 99.98% sensitivity rate and 99.95% precision rate for the Narrow NN classifier. The other classifiers also obtained improved accuracy. The confusion matrix in Fig. 11 verifies the obtained performance measures. The correct prediction is illustrated by diagonal values in the confusion matrix. It is further noted that the feature fusion step increased the performance in terms of accuracy and precision rate, whereas the computational time is also increased.

**Table 9:** Classification results after employing the proposed fusion technique.

Classifier	Accuracy (%)	Time (s)	Sensitivity rate (%)	False negative rate (%)	Precision rate (%)	Area under curve (%)
Narrow NN	**100**	**156.692**	**99.98**	**0.02**	**99.95**	**1.00**
Medium NN	100	84.31	99.95	0.05	99.95	1.00
Wide NN	100	175.46	99.95	0.05	99.95	1.00
Bi-Layered NN	99.90	258.998	99.95	0.05	99.93	1.00
Tri-Layered NN	99.90	305.48	99.85	0.15	99.85	1.00

Significant values are in bold.

**Figure 12:**
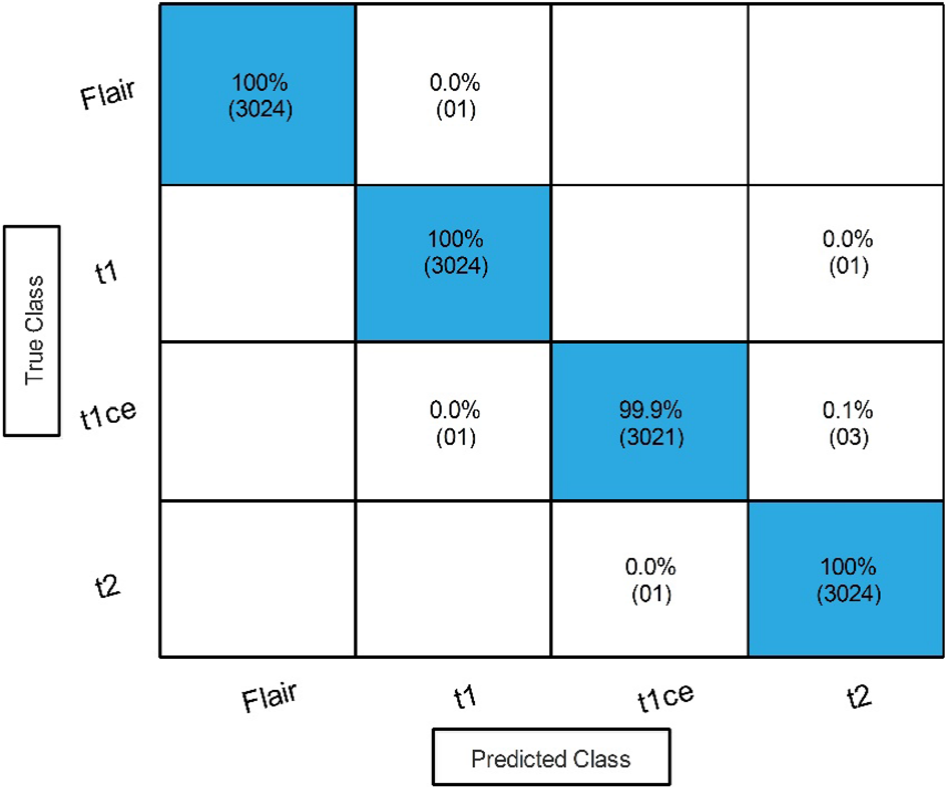
Confusion matrix of improved version of Grey Wolf Optimization on BraTS2021 dataset.

**Figure 13:**
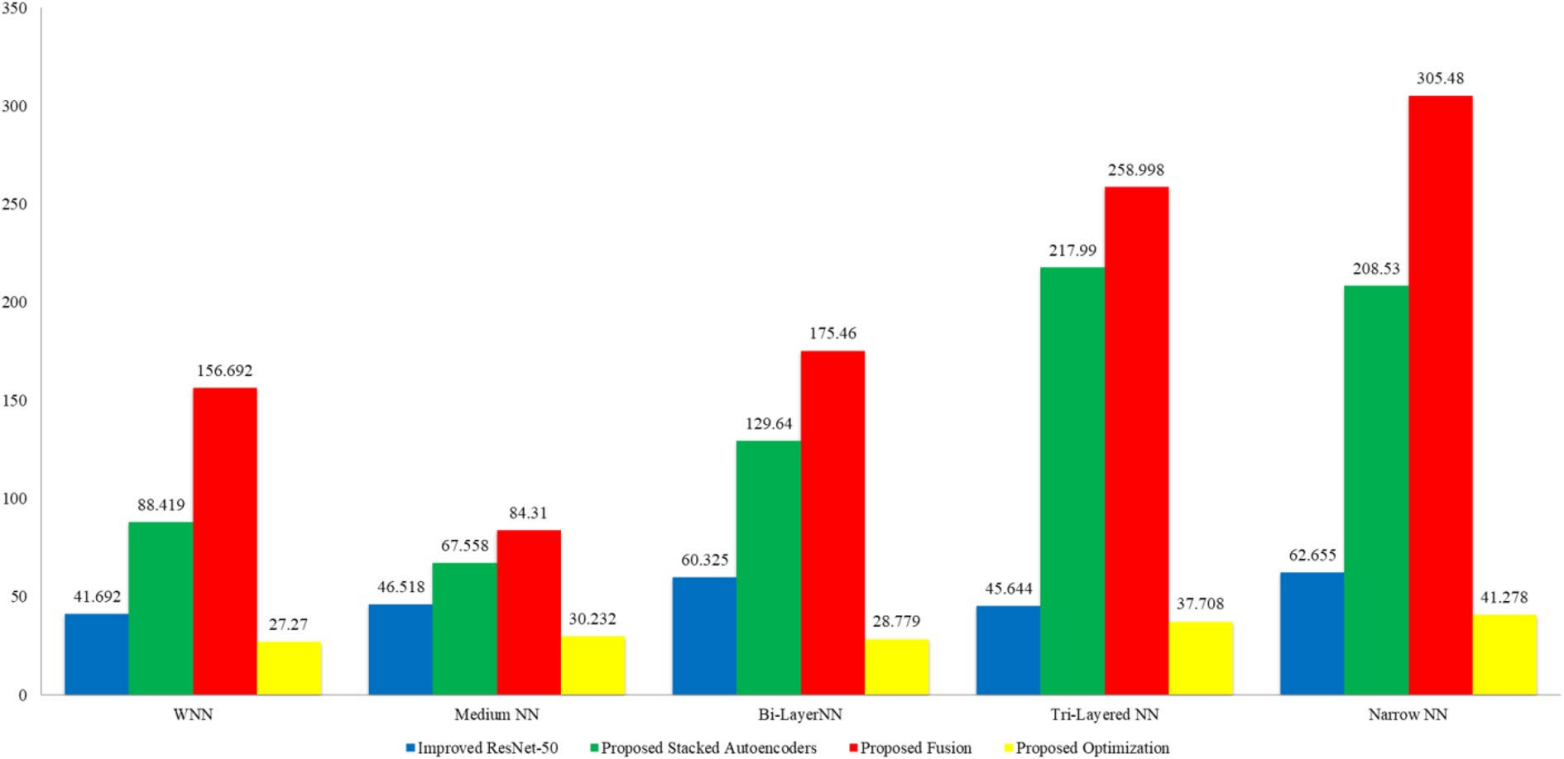
Comparison among all middle steps based on the computational time for BraTS2021 dataset.

In the last step, an improved version of the Grey Wolf optimization technique is employed, and the results are described in Table 10. The result describes that 99.90% best accuracy is noted along with 99.98% sensitivity rate and 99.95% precision rate for the Narrow NN classifier. Moreover, an accuracy of 99.90% was noted for Bi-Layered Narrow and Wide NN classifiers, while 99.80% accuracy was noted for Tri-Layered NN classifiers. In addition, the minimum noted time is 27.270 (s) for Medium NN. Figure 12 verifies the obtained performance measures. The correct prediction is illustrated by diagonal values in the confusion matrix. The accuracy of this phase is almost equal, but the computational time is significantly reduced, which is a strength of this work. A time comparison for this dataset among all middle steps is shown in Fig. 13. This figure shows the strength of the improved version of Grey Wolf Optimization.

**Table 10:** Classification results after employing an improved version of Grey Wolf Optimization.

Classifier	Accuracy (%)	Time (s)	Sensitivity rate (%)	False negative rate (%)	Precision rate (%)	Area under curve (%)
Medium NN	**99.90**	**27.270**	**99.98**	**0.02**	**99.95**	**1.00**
Bi-Layered NN	99.90	30.232	99.95	0.05	99.95	1.00
Narrow NN	99.90	28.779	99.93	0.07	99.93	1.00
Wide NN	99.90	37.708	99.93	0.07	99.93	1.00
Tri-Layered NN	99.80	41.278	99.83	0.17	99.83	1.00

Significant values are in bold.

### Student's T-test based analysis

We have performed this test in order to validate our null hypothesis for both datasets, that is given as follows: ${h}_{0}$= There is a significant difference between the accuracies of selected classifiers. In order to perform our experiments, firstly, we have selected the two classifiers with corresponding accuracies in all experiments for a dataset of BraTS2020 dataset. One classifier which is performing with best accuracy among all experiments and the other classifier which is at the lowest level of performance among all the classifiers. The detail is given in Table 11.

**Table 11:** Classifiers with relevant accuracies for all experiments (BraTS2020).

Classifier	ResNet-50	Stacked autoencoders	Fusion process	Grey Wolf & Jaya optimizers
Wide neural network	99.70	98.90	99.90	99.80
Narrow neural network	99.70	92.70	99.90	99.60

The mean of the differences for all experiments are were calculated using following equations:

20 $$Difference \left(D\right)= \left|Accuracy(i)-Accuracy(j)\right|$$

$$Accuracy \left(i\right)= Accuracies\, of\, first\, classifier$$

$$Accuracy (j) = Accuracies\, of\, second\, classifier$$

21 $$Mean (\mu )= \frac{1}{N}\sum_{i=1}^{N}\left|{D}_{i}\right|$$

where $N$ is the number of experiments and the noted mean value after this step is $1.6$. After calculating the $Mean (\mu )$, the $standard\, deviation (\sigma )$ is calculated by using following equation:

22 $$standard\, deviation \left(\sigma \right)= \sqrt{\frac{{(\sum_{i=1}^{N}({D}_{i})-\mu )}^{2}}{N-1}}$$

The resultant standard deviation value is $3.065$ later used in the $T-Selection$ formula.

23 $$T-Selection=\frac{\sqrt{N }\times \mu }{\sigma }$$

The resultant value of this equation is $1.044$. This value will be considered as a reference point to conduct the *Student’s T-Test*. In our next step, we find the degree of freedom as $Degree of Freedom\left(df\right)=n-1$; hence, the $df$ value is 3 and $p value = 0.05$^53^. In the t-table, the output value is $(-3.182, +3.182)$. As the t-selection value for this dataset is 1.044, based on the given below formulation, this hypothesis is rejected, and there is no significant difference in the accuracy of the two classifiers for all methods.

24 $$If (T-Selection >= -3.182 \,and <= + 3.182)$$

Similarly, this process is conducted for BraTS2021 and obtained the t-value of 1.5, $df$ is 3, value of t in the table is $(-3.182, +3.182)$ at p-value 0.05. The t-value is not fall under this interval; hence, the hypothesis ${h}_{0}$ is rejected.

### Grad-CAM based performance analysis of improved ResNet-50

The classification score depends upon the gradients; Grad-CAM utilizes it with respect to the obtained final convolutional feature map. This visualization method highlights the areas of the input image with a higher contribution to the classification score. The features are extracted from global average pooling layer. This method works using Eq. (25), which is given below:

25 $${a}_{k}^{c}= \frac{1}{N} {\sum }_{i}{\sum }_{j}\frac{{\partial }_{y}^{c}}{{\partial A}_{i,j}^{k}}$$

where ${a}_{k}^{c}$ are class scores of $k$ features of the Global Average Pooling layer, $N$ represents total pixels in a feature map, $c$ depicts the class score, and $y$ is considered as output. The whole expression ${\partial A}_{i,j}^{k}$ represents the convolution map. In the expression, $i$ and $j$ represent two dimensions, and $A$ represents gradients. The output of this equation may be negative feature weights; therefore, a ReLu activation function is employed to resolve this issue.

26 $${\text{M}}={\text{Relu}}\left(\sum {a}_{k}^{c} . {A}^{k}\right)$$

**Figure 14:**
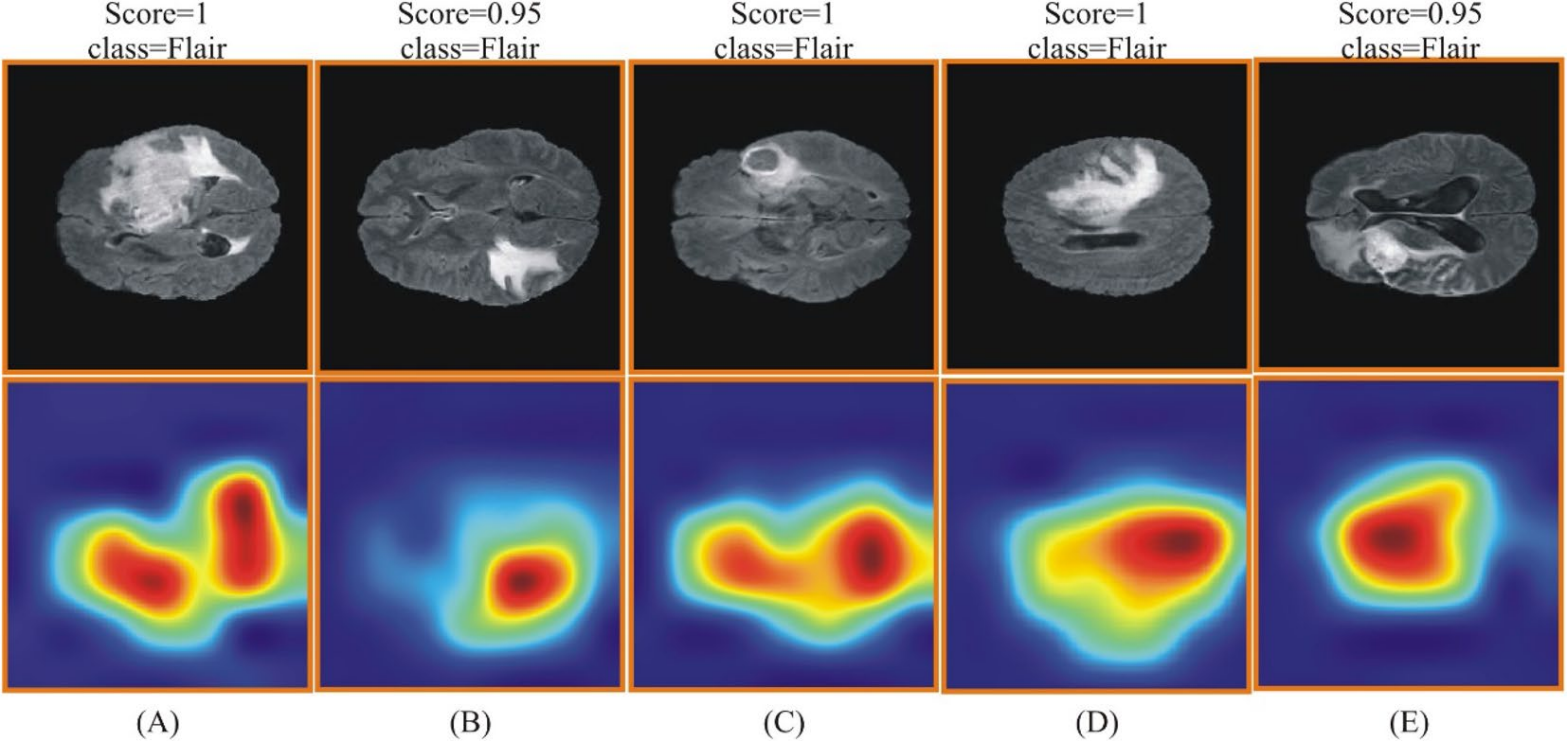
Grad-CAM based heat map for BraTS2020 dataset using improved ResNet-50. (**A**–**E**) showing the original and heat map images.

**Figure 15:**
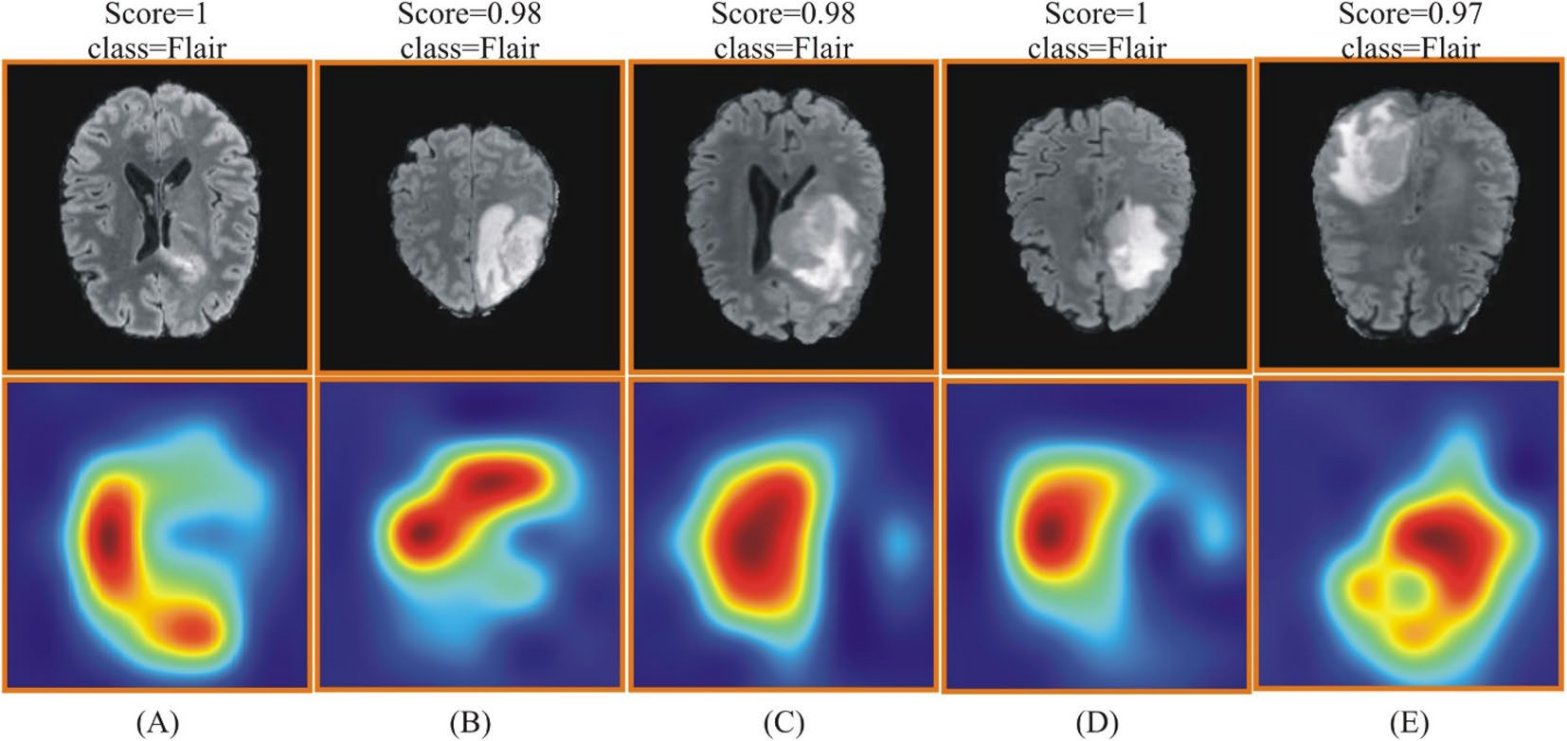
Grad-CAM based heat map visualization for BraTS 2021 dataset using improved ResNet-50. (**A**–**E**) showing the original and heat map images.

The visual results of this method are shown in Figs. 14 and 15. These figures show that the score and class output are returned against each image as a predicted label. The highlighted region is the most important region utilized for the corresponding label.

**Figure 16:**
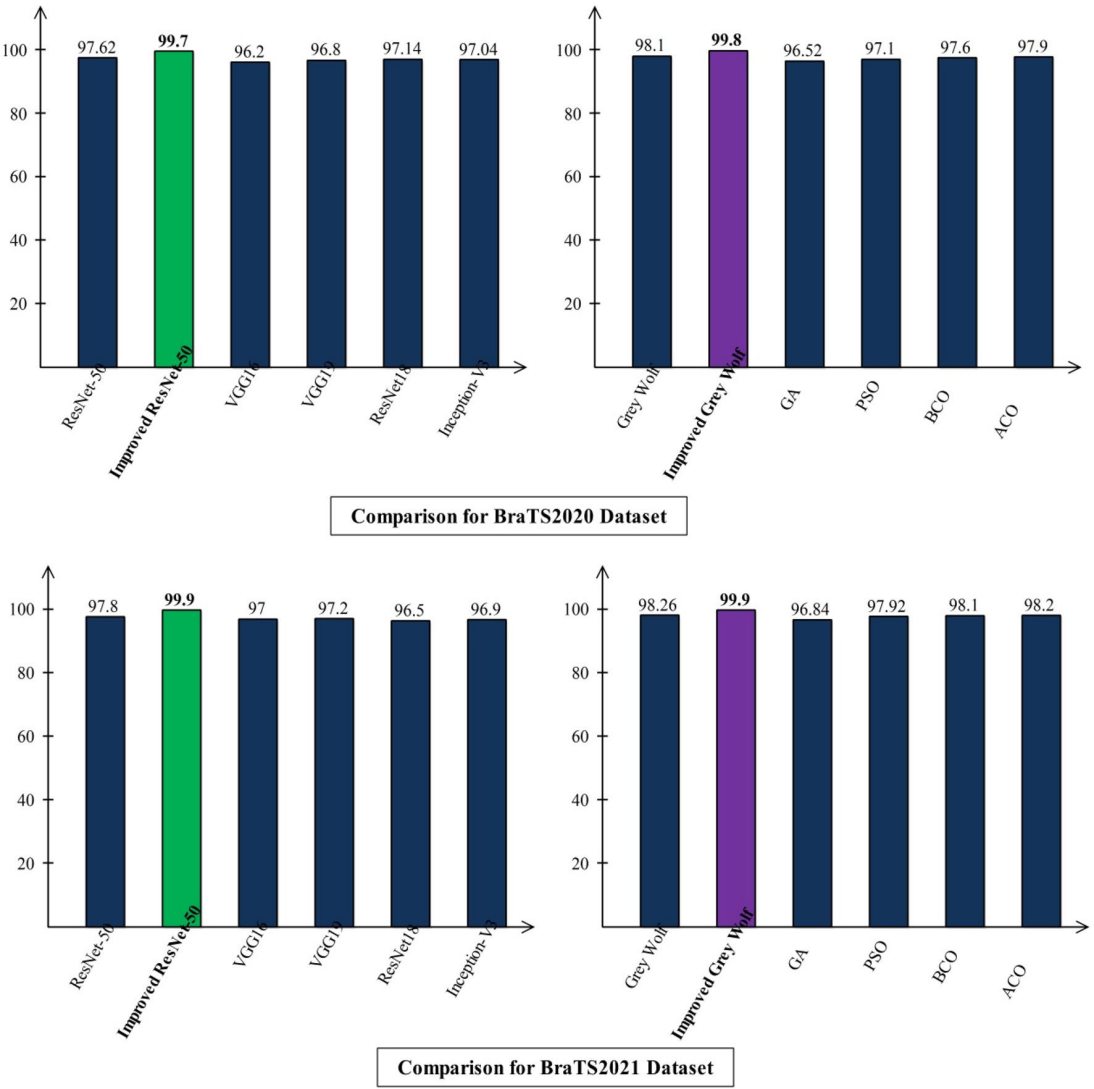
Comparison of original and improved CNN and optimization methods in terms of accuracy.

After the visualization, a short discussion is conducted based on graphical results. The discussion is conducted for three different scenarios: (i) Original ResNet-50 vs. Improved Version of ResNet-50; (ii) comparison of improved ResNet-50 with several other neural nets; and (iii) Original Grey Wolf vs. Improved version of Grey Wolf and with several other methods. Figure 16 shows the results of these scenarios. This figure clearly illustrates that the improved version accuracy is better than the original neural nets and optimization algorithms. Lastly, a comparison is also conducted with recent state-of-the-art techniques, as described in Table 12. This table shows that the proposed method obtained improved accuracy than the recent techniques on the selected datasets.

**Table 12:** Comparison of the proposed accuracy with SOTA techniques.

Reference	Year	Dataset	Accuracy (%)
Gull et al.^54^	2022	BraTS2020	98.25
Farajzadeh et al.^55^	2023	BraTS2020	98.93
Yadav et al.^56^	2023	BraTS2020	99.46
Ferdous et al.^57^	2023	BraTS2021	93.69
Proposed		BraTS2020	**99.80**
		BraTS2021	**99.90**

Significant values are in bold.

## Conclusion

The research of medical image applications that employ deep learning and optimization strategies and adhere to the concept of effective natural images in practical applications has attracted more attention in recent years. MRI data has been frequently used with deep learning techniques for automatic brain tumor detection and classification. This paper provides a fully automated computerized multiclass brain tumor classification system with deep learning and optimization. In the proposed architecture, an improved ResNet-50 model is designed and automatically initializes the hyperparameters using the Jaya algorithm that is later employed to learn a model. In addition, a new Stacked Autoencoders network is designed and trained on the selected dataset. Features are extracted and fused using a novel fusion technique, showing improved accuracy. However, a few redundant features have been detected that were further removed using an improved version of the optimization technique. The experimental process is conducted on two publicly available datasets and obtained with improved accuracy. The improved version of the ResNet-50 model improved the classification accuracy and reduced the number of total parameters. In addition, the fusion process improved the accuracy, but time is increased. To address this challenge, a feature selection technique is proposed that maintained the accuracy and almost 70% reduced the computational time.

## Data Availability

The data that support the findings of this study are openly available at: https://www.med.upenn.edu/cbica/brats2020/ and https://www.med.upenn.edu/cbica/brats2021/.
